# Water and national identity in the Netherlands; the history of an idea

**DOI:** 10.1007/s12685-020-00263-3

**Published:** 2020-11-17

**Authors:** Erik Mostert

**Affiliations:** Department of Water Management, Delft University of Technology, Stevinweg 1, 2628 CN Delft, The Netherlands

**Keywords:** Netherlands, Water, National identity, Culture, Water boards, Democracy

## Abstract

According to a popular Dutch theory, water has shaped the Dutch national identity. The Dutch fight against the water would have stimulated perseverance, ingenuity, cooperation and an egalitarian and democratic society. Despite the long water management history of the Netherlands, water became an important part of the self-images of the nation only in the eighteenth Century. In the 1780s the idea that the Dutch had wrung their country from the sea became popular. Initially, this idea was especially popular among the (proto-)liberal opposition, who emphasised the importance of the nation and its achievements. By the end of the nineteenth Century, water had become a national symbol for orthodox Calvinists, Roman Catholics and Socialists too, despite their different views on the nation. Whenever there was fast social change, political turmoil or external threats, as in the late eighteenth Century, the 1930s and 1940s and since the 1990s, the link between water and the Netherlands was used to promote national pride and unity and stimulate action. This link has also been used to promote specific hydraulic works, but it is a topic for further research how widespread and effective this practice was. As this paper is part of a special issue, Water History in the time of COVID-19, it has undergone modified peer review.

## Introduction

According to a popular Dutch theory, water has shaped the Dutch national identity. Due to its geography, the Netherlands have been in a constant struggle to protect the country from flooding and reclaim land. This struggle required perseverance, ingenuity and cooperation. Especially the latter would have resulted in an egalitarian society and democratic institutions, the oldest being the water boards (e.g. Vuijsje and Lans 1999; Pleij 2016; Van den Brink 2018; Kullberg et al. 2019).

This theory is interesting from an international perspective as well. In different countries and at different times, water resources development has been advocated as a means for national development or regeneration, for instance in Spain from the late nineteenth Century onwards (Swyngedouw 1999) and in Ethiopia, where the “Grand Ethiopian Renaissance Dam” is currently being built. The Dutch theory goes further. It asserts that the nation and its institutions are the result of water resources development or, as the Dutch lawyer Donner put it, “the State, that are the dykes” (Van der Pot and Donner 1977, p. 138). In his book *Oriental despotism*, Wittfogel (1957) attributed an equally large role to water resources development, but he argued that large hydraulic works stimulated despotic regimes rather than democracy.^1^

The aim of this article is to explore the link between water and national identity, using the Netherlands as an example. National identity can be defined either in essentialist terms, as the objective characteristics of a group that shares a common descent, language, values and/or religion; or in social-constructivist terms, as the self-images of a discursively constituted “nation” (e.g. Kesic and Duyvendak 2016; Leerssen 2016). In this article, I use national identity primarily in the second sense, as self-images. These images are cultural rather than individual. They are influenced by and in turn influence the educational system, politics and the mass media. They develop in a specific historical and political context (Leerssen 2011, p. 20) and establish the nation as an “imagined community” (Anderson 2006), determining who are part of nation and who are not. To be convincing, they often refer to facts, such as the large presence of water in the Netherlands, but these facts are always used selectively and they do not always stand up to close scrutiny.

The purpose of this article is to describe when water started to figure in discourses on the Dutch national identity and how this role has developed since. The sources I use for this are different expressions of Dutch culture, such as Dutch literary works, newspaper reports and historical works, the latter both as a primary source for the time they were written and as a secondary source for the period they discuss. Many of these sources are available online on Delpher (www.delpher.nl), in the Digitale Bibliotheek voor de Nederlandse Letteren (www.dbnl.nl) and on YouTube. To find relevant texts, I used search terms such as “strijd tegen het water” (fight against the water) and “nationaal karakter” (national character). In addition, I used the snowballing technique, checking potentially relevant references in the texts found, the references in these references, and so on. From the many texts found, I selected those that expressed a pertinent notion for the first time or were quite popular at the time, e.g. were reprinted often.

In this article, the different elements of the current theory on water and the Dutch national identity are discussed in a more-or-less chronological order, starting with a section on the Dutch fight against the water. This section also provides a succinct overview of the development of the Netherlands as a nation, since without a nation there cannot be a national identity. The following sections go more into detail concerning floods, land reclamation, national virtues and the water boards. In addition, shipping and trade are discussed briefly since these topics have been linked to both the fight against water and the national identity. The penultimate section discusses emotional attachment to water as a landscape feature. The final section addresses the question how important water has been for the Dutch national identity and gives recommendations for further research.

## The fight against the water and the Dutch nation

The oldest element of the theory on water and the Dutch identity, predating the Dutch nation by several centuries, is the fight against the water (“strijd tegen het water”). The fight against the water has often been likened to the fight against human enemies. According to the Rüstringer law, an old Frisian law from around 1300, “we (…) have to protect our land against the sea using three instruments: the spade, the burry and the fork. Moreover, we (…) should protect our land with sword, spear and brown shield (…) against unlawful dominion” (Buma and Ebel 1963, p. 90). In the play Gijsbrecht van Aemstel by Joost van den Vondel from 1638, to give just another example, the City of Amsterdam is besieged by the Count of Holland, and when the Count’s men force entry into the city, they are compared to a “wintery storm that threatens the dyke (…) and proves to be too strong” (Van den Vondel 1994, lines 1190–1193).

The fight against human enemies often received far more attention than the fight against the water (cf. Jensen 2016). This may be explained by the fact that the Dutch nation is often seen as the result of political struggle. At the time of the Rüstringer law, the Netherlands did not yet exist as a political or cultural entity. Instead, there were a number of separate territories. By the mid-sixteenth Century, these had all been inherited by the Habsburg kings of Spain, but in 1568 a revolt against the Spanish rule broke out, led by William of Orange, because the taxes had been raised, local privileges were violated and Protestants were prosecuted. The revolting territories joined forces and together they established the “Republic of the Seven United Netherlands”.

The Republic was a loose federation with a common army and navy and a common foreign policy, but most other competencies remained at the regional or local level. There was, however, quite some mobility within the Republic and by the 1730s a national “communication community” had developed as a result of increased publishing and reading (Van Sas 2005, p. 72).^2^ In this community, the decline of the Republic since the seventeenth Century was lamented and there were many calls for moral regeneration. Around 1780, these calls turned political, when the so-called Patriots challenged the rule by the Oranges and a limited number of patrician families, and in 1787 a revolution broke out. This revolution was, however, suppressed. After a period of increasing French influence (1795–1810) and incorporation into the French State (1810–1813), the Netherlands became a Kingdom under the House of Orange in 1813. Yet, in the end, the Patriots, reincarnated as liberals, won: in 1848 the Netherlands got a liberal constitution. After some final political skirmishes in the 1860s, they even embraced the Oranges as a national symbol (Bank and Buuren 2000).

In the nineteenth Century, there were not only liberals in the Netherlands, but also many orthodox Calvinists, Roman-Catholics and, after 1860, Socialists.^3^ The orthodox Calvinists opposed the principle of popular sovereignty and saw the Netherlands as a Calvinist nation led by the House of Orange. According to one of their leaders, Providence would have “linked the glory of Holland with the glory of a National Dynasty, and the welfare of both to Religiosity and Religious freedom” (Groen van Prinsterer 1830, p. 91; see also Kuyper 1879, art. 1 and 2). The Roman-Catholics had been marginalised under the Republic as they were not allowed to hold public office or worship in public. Initially, they did not have a strong national identity and were focused mostly on the local parish and on Rome, but later in the century they claimed the pre-reformation history of the Netherlands (Raedts 2011, pp. 249–261). When in 1898 a new Queen was inaugurated, they participated actively in the festivities (Bank and Buuren 2000, p. 27). The Socialists had an international outlook and were against the monarchy.

In the first half of the twentieth Century, the Netherlands had become a highly “pillarised” nation, with a Calvinist, Roman Catholic, Social Democratic and “neutral” pillar, each with its own political party, newspapers, broadcasting corporation, schools and trade unions. Yet, each pillar saw the Netherlands as one nation. In the 1930s and the 1940s, there was even an upsurge in attention for the Dutch national identity, as witnessed by the many booklets on the “national character” (e.g. Kruijt 1934; Josephus Jitta 1936b; Stibbe 1940; Romein 1942; Rüter 1945; Staverman 1946; Van Heerikhuizen 1980) and the many books on Dutch culture and the Dutch landscape (e.g. Brusse et al. 1935; Werumeus Buning 1940; Huf 1941; Korevaar-Hesseling 1941; Van Leeuwen 1941; Donker 1945; NN 1950). This upsurge can be linked to the increasing threat from Nazi Germany and the Second World War (cf. Romein 1942, pp. 16–17).

In the 1960s, the Netherlands rapidly became more secular and the different pillars slowly disintegrated. For several decades, talk of a national identity was suspect, as the concept was seen as parochial and was associated with extreme right-wing ideologies (e.g. Herman Pleij in Van Eijndhoven and Leverink 2009). This changed in the early 1990s, when the importance of the Dutch history and Dutch culture was emphasised again (cf. the opinion article of Frits Bolkestein in the *Volkskrant* of 12 September 1991). This renewed attention could be explained by unease about large-scale immigration since the 1960s, but also as an effect of the dissolution of traditional bonds and a reaction to neo-liberal politics with its emphasis on privatisation and competition.

## Floods

In the fight against the water there were successes as well as setbacks. From the seventeenth Century onwards, many accounts of floods were published. Originally, they were often presented as Divine punishments and warnings for sins committed, such as discord, luxury and lack of faith (Alutarius 1656–1657; De Vries 1687; Schama 1988; Sundberg 2015; Van Asperen 2020). In somewhat less orthodox accounts, they were (and to some extent still are) used to show God’s grace, for instance when flood victims are saved miraculously or welcomed in Heaven (see Mostert 2015).

Floods were often linked to the monarchy (Jensen 2018). From 1809 onwards, the reigning monarch has always visited flood disaster areas to offer moral and sometimes material support (Bosch 2012). Moreover, orthodox Calvinist saw the hand of God in both floods and the monarchy. When, for example, King William III celebrated his silver jubilee in 1874, the prints and books commemorating the occasion nearly always included a picture of the king visiting a flood disaster area, usually combined with pictures referring to the revolt against the Spanish or the Netherlands regaining independence in 1813, and sometimes with Bible texts (e.g. Andriessen 1874; NN 1874, Fig. 1; cf. Mostert 2015).

**Fig. 1:**
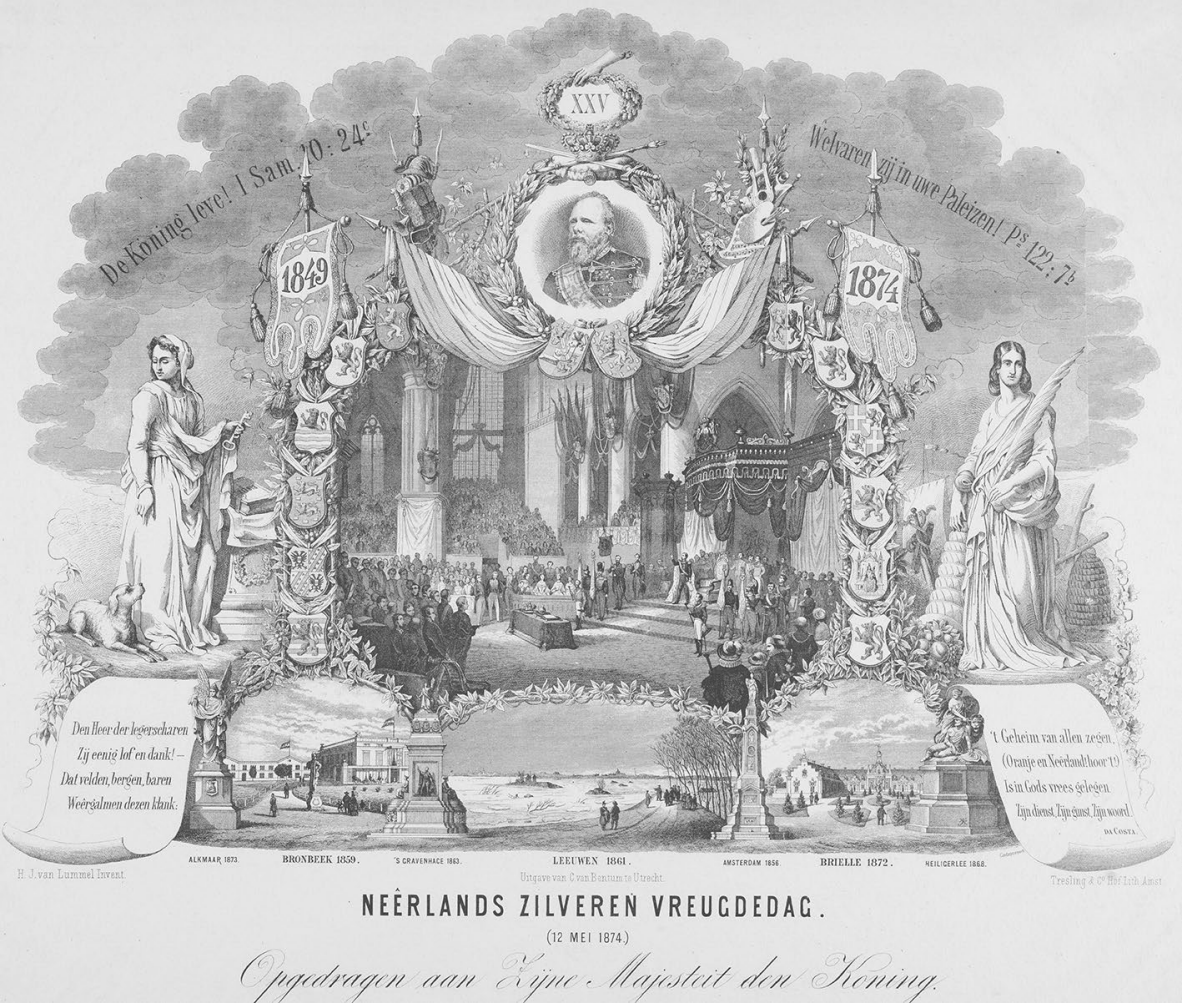
God, the Netherlands and Orange—and floods. Print commemorating King William III’s Silver Jubilee, showing his visit to the flooded village of Leeuwen in 1861 (bottom centre; Collection Rijksmuseum, Amsterdam, inv. nr RP-P-OB-89.241)

Floods did not only stimulate religious and monarchist feelings, but also feelings of solidarity and practical relief work (Duiveman 2019). Traditionally, relief work was organised locally or in nearby towns, but from the eighteenth Century onwards it was organised increasingly at the national level. The first example is the 1740–1741 flooding in the Dutch river area, when citizens of Rotterdam, Amsterdam and other cities collected money and goods and organised transport to the disaster area (Bosch 2012). The motivation given by one of the organisers, Jan Wagenaar, was that “they felt the need of their compatriots from afar” (Bosch 2012, p. 42). Especially the word compatriots (“landgenoten”) suggests that by 1740 the Dutch nation in the sense of an imagined community already existed, at least in the eyes of the urban middle classes.

Large flood disasters continue to stimulate feelings of national solidarity. When in January–February 1995 nearly a quarter of a million people had to be evacuated because of a threat of flooding, a large television show was broadcasted and a commemorative book was published to raise money for the evacuees. In the foreword to the book, the Prime Minister expressed his appreciation for the “sense of community” and “solidarity” that the Dutch people had shown (Vandersmissen et al. 1995).

## Land reclamation

Land reclamation constitutes the successes in the Dutch fight against the water. For centuries, the inhabitants of what is now the Netherlands have been draining swamps and reclaiming lakes and coastal floodplains (Van de Ven 2004). Yet, until the late eighteenth Century, there is no clear evidence to suggest that they derived their national identity from these activities. For example, in 1644 the Dutch poet Joost van den Vondel wrote an elegy on the reclamation of the Beemster Lake, in which he compared the newly created polder with Aphrodite, born out of the foam of the sea (Van den Vondel 1930). He saw the reclamation as the conquest of civilisation over uncivilised nature and order over chaos (Fleischer 2007), but there is no reference to a Dutch national identity—which is not surprising as the Dutch nation had not yet fully developed.

From the late eighteenth Century onwards, many Dutch prided themselves on the fact that they had created their own country. This pride was expressed by the saying “God created the world, but the Dutch created the Netherlands”, first recorded in 1818 (NN 1818, p. 186), but derived from an older verse in Latin (Pitcairn 1727, p. 168; see Janiçon 1729, p. 9, 1731, p. 13; Van Haren 1776, p. 168; Feith 1793, p. 47; Niemeijer 2016). More commonly, this pride was expressed by the word “ontwoekerd”. “Ontwoekerd” means wrung from, and it was used primarily in the sense of turning water or a swamp into productive land. In this sense, it is first recorded in 1783 (Al–Z 1783, p. 265). One year later, it was used by the popular authors Wolff and Deken (1784, p. 329). It is also used in the famous poem “Holland” by Potgieter from 1832, of which a translation is given Box 1.

Box 1: Holland, by E.J. Potgieter (1833, own translation)

**Table:** 

Grey is thy sky and stormy thy beach,
Naked thy dunes and even thy fields,
Thou created nature with a stepmotherly hand,–
Yet, I love thee dearly, O my Land!
All that thou art is the work of the Fathers;
From a swamp did the diligence of these heroes,
Too strong for the sea and the tyrant alike,
Wring Freedom a temple and Piety a church.
Stay what thou were when thou shone like a flower:
Ensure that Europe calls thee the seat of order
And the oppressed his refuge,
Land of my Fathers, my joy and my glory!
And what the dark future has in store,
Whatever its pregnant clouds may bear,
Laurels belong to the unblemished sword,
Fatherland! once the freest on earth.
In Sweden. 1832.
E.J. Potgieter.

Similar ideas are expressed in the often reprinted poem *The Dutch nation* by Helmers from 1812. “Which people”, he writes, “has more right to praise their ancestors? The soil their offspring walk on, each must call their work” (Helmers 2009, p. 110). Helmers presented reclamation as a peaceful method of conquering land: “Never, never o Fatherland! did you take up arms to take someone else’s land to extend your boundaries” (p. 202). In later years, reclamation was presented as a peaceful activity as well (e.g. Van der Houven van Oordt 1898). This fitted very well in the self-image of the Netherlands as a peace-loving country that is provoked only when its freedom and independence are threatened (e.g. Stibbe 1940).

“Ontwoekerd” soon became a cliché, used in passing to express and stimulate love for one’s country (e.g. Feith 1793, p. 23; Van Lennep 1828, p. 8). It is used twice in a parody of nineteenth Century poetry extolling virtue, piety and patriotism (Paradijs 1887, pp. 16, 61). Only occasionally the idea that the Dutch had created their own country was challenged. According to the conservative author Willem Bilderdijk, the construction of dykes had stopped natural sedimentation and the reclamation of lakes had reduced water storage capacity, both aggravating flooding problems (Bilderdijk 1832, p. 24; similarly Multatuli 1877, idea 1050d). For some orthodox Calvinists, the idea bordered on blasphemy. According to them, God had founded the Netherlands, and while the Dutch had reclaimed a lot of land, God reminded them, by means of fearsome floods, of their dependence on Him (Groen van Prinsterer 1841, p. 2, 1846, pp. 3–4).

Land reclamation was initially especially popular among the Patriots (e.g. Wolff and Deken 1784; Feith 1793; Moens 1827). The Patriots emphasised the importance of the nation, the people of the Netherlands themselves, and presented land reclamation as one its main achievements. Later, land reclamation became popular among Calvinists and Socialists as well, but the Calvinists emphasised that it required God’s blessing (e.g. Grimme 1939; Norel 1939), and the Socialists stressed that it could only fully benefit the country if the economic system would change (e.g. Last 1939 and the documentary Nieuw gronden/New earth by Joris Ivens from 1933, available on YouTube).

Currently, land reclamation sometimes has a conservative flavour (e.g. the YouTube video “Het land van FVD” by the Dutch anti-EU and anti-immigration politician Thierry Baudet). It is part of the so-called “historical canon” of the Netherlands, which was developed in 2005–2006 for use in schools to provide the students with the basic knowledge of Dutch history and culture that all Dutch citizens should possess (Committee for the Development of the Dutch Canon 2007). The canon consists of fifty chronologically ordered themes or “windows”, including the reclamation of the Beemster Lake (https://canonvannederland.nl).

## Shipping and trade

Shipping and trade do not figure prominently in the current theory on water and the Dutch identity, but they are often seen as very important for the Dutch identity. Moreover, they have been linked with flooding and land reclamation. Because of the small size of the country and the humid conditions, employment possibilities in agriculture were limited, but the possibilities for fishing, shipping and trade were excellent. Hence, many turned to these occupations (Ockerse 1797, pp. 25–27; Hora Siccama 1848, pp. 47, 52–53; Borger 1997; De Neve and Van Heezik 2007). Around 1600, the Netherlands started to establish trading posts and colonies overseas, such as the Dutch East Indies (now Indonesia), and together with trade within Europe, this brought vast wealth to the country. This was partly invested in land reclamation projects. Along with the development of shipping and trade, the Netherlands also set up a large navy, which won many celebrated battles in the seventeenth Century.

When the Dutch historian Huizinga referred to the importance of water for the Dutch civilization in the seventeenth Century, he referred primarily to shipping and trade. These would have given the Netherlands an urban and commercial character without a clear class structure (Huizinga 1941, pp. 21–23). There is a plethora of cultural references to shipping and trade in the Netherlands. In the seventeenth Century, Joost van den Vondel wrote two long poems “in praise of shipping” (Van den Vondel 1987), in the early nineteenth Century Helmers wrote extensively about shipping and the Dutch involvement in the East Indies (Helmers 2009), large funeral monuments for (vice-)admirals were erected (Lawrence 2016) and many streets have been named after them, and many children’s books deal with shipping, including classics such as “Paddeltje” (Been 1908), reprinted more than 50 times. The historical canon of the Netherlands also includes several windows related to shipping and trade, such as the windows on the Dutch East Indies Companies and admiral Michiel Adriaensz de Ruyter.

## National virtues

Both the fight against the water and shipping and trade would have given the Dutch people specific personal characteristics. The first author to develop this theory in some detail was the radical politician Willem Anthonij Ockerse in 1788–1797 (Van Sas 2005, pp. 303–313). According to Ockerse, the waterlogged conditions in the Netherlands and the threat of flooding had required the first inhabitants to work hard and develop expertise in building dykes and constructing wind mills. In turn, this had stimulated industry and the arts and sciences, in particular practical sciences such as mathematics and engineering (Ockerse 1797, pp. 28–37; see also Engelberts 1763, pp. 35–36). Since the land could not provide the Dutch with everything they needed, they naturally turned to fishing and shipping, and in the seventeenth Century established colonies overseas (Ockerse 1797, pp. 25–27). These activities brought vast wealth to the Netherlands, but they also created a taste for luxury and increased foreign influence, which weakened the original character of the Dutch (pp. 41–42, 47–48).

The humid conditions in the Netherlands would also have a direct effect on the national character: it made the Dutch people phlegmatic. They were presented as slow, careful, thoughtful, difficult to excite and influence, and persevering, but unlikely to show flashes of genius; more the type of a deep and diligent thinker (Ockerse 1797, pp. 43–45; see also Sturkenboom 2014). In addition, the humid and unhealthy conditions stimulated the proverbial cleanliness of the Dutch women concerning their house (p. 63–67). Other authors have explained this cleanliness by its association with moral purity (Schama 1988, pp. 379–400), and by the need for hygiene in dairy making, which was widespread in the Netherlands (Pleij 2016, pp. 133–134).

The characteristics discussed by Ockerse can also be found in many later descriptions of the Dutch national character (Meyer 1830; Hora Siccama 1848; Stibbe 1940; Pleij 2016; Van den Brink 2018). Stimulated by the natural conditions, the Dutch would be hardworking, resilient and stubborn, ingenious, and entrepreneurial. Sometimes they are also presented as religious, which is then explained by the precarious nature of their existence (Hora Siccama 1848, p. 45). While the term phlegmatic is rarely used anymore, the Dutch are still described as down-to-earth (“nuchter”) and not very emotional, with occasional emotional eruptions that would only confirm their generally down-to-earth character (Fruin and Vissering 1900; Josephus Jitta 1936b; Donker 1945; Pleij 2016).

In addition, the geography of the Netherlands would have stimulated self-reliance, independence and a love of freedom. According to some accounts, the first inhabitants of the Netherlands were refugees from more richly endowed countries, who developed their own ways to develop and live from the land (Meyer 1830). The many man-made ditches and later military inundations helped to defend the country against foreign invasion, and feudalism never really got a hold (cf. Huizinga 1941, p. 23; Van Schendel 1945).

A moot point is the issue of cooperation. Many authors argue that the fight against water was a huge task that required the cooperation of many. This would have stimulated cooperation in many other fields as well (e.g. Van den Brink 2018; Pleij 2016). According to others, however, cooperation was motivated primarily by the self-interest of the land owners and did not extend beyond their own dyke (e.g. Blink 1896, p. 555; Kruijt 1934, p. 60, 1938, p. 17; Dibbits 1950, pp. 88–91). Wider cooperation often had to imposed by a strong central government (Romein 1934, pp. 28–29).

## The water boards

The most popular Dutch theory nowadays is that the fight against water did stimulate cooperation. This resulted in an egalitarian society and democratic institutions, the oldest being the water boards. Water boards are the regional and local water management bodies in the Netherlands. Regional water boards date from the twelfth Century onwards and initially had supervisory powers over the local communities and individual land owners responsible for the different water management works. From the late Middle Ages onwards they increasingly executed these works themselves, financed by levies. The local water boards were established from the late Middle Ages onwards to finance local water works, such as a polder mill to drain a polder. In the twentieth Century, the local water boards merged with the regional water boards, of which there are currently 21 left (e.g. Mostert 2017).

The water boards were not always seen as democratic. The Patriots of the late eighteenth Century saw them as part and parcel of the aristocratic system of governance by the Oranges and a limited number of Patrician families (Ultrajectinus 1783; Voorregt 1784; Van der Ham and Jacobs 2004). Half a century later, the liberals considered the larger water boards, with their combination of executive, legislative and judiciary powers, as remnants of the *ancien régime* in dire need of modernisation (Donker Curtius 1834, 1835; Potgieter 1844, p. 644; Stuurman 1992, pp. 124–126).

The first author developing the notion of water boards as the oldest democratic institutions was the lawyer Meyer (1820). According to Meyer, the water boards were the first communities in the Netherlands. The first inhabitants of the country would soon have realised that they had a common interest on top of their individual interests and had to cooperate in their fight against the water (pp. 23–24). This resulted in a cooperative spirit that spilled over into other spheres of public life as well (pp. 26–27). As cooperation required leadership and expertise, the interested inhabitants established local water boards that derived their authority exclusively from the inhabitants themselves (Meyer 1830, pp. 68–71). Unlike many other communities, however, the water boards were motivated by material interests only and could therefore be seen as associations of properties rather than of people (Meyer 1820, p. 31).

While Meyer’s theory was mostly forgotten,^4^ a new theory was developed in the 1930s by Josephus Jitta (1932, 1936a). Josephus Jitta was interested in democratic alternatives to the State and the market for ordering economic life. The water boards would be the oldest and typically Dutch example of such an alternative, which he called functional decentralisation. Functional decentralisation refers to the establishment or recognition by government of organisations of experts and stakeholders with a common, usual material interest that perform specific government functions, such as issuing advice and implementing policies. According to Josephus Jitta, such organisations can be very effective means of governance, provided legislative bodies or the executive representing the general interest remain in control.

When the autonomy of the water boards started to be challenged in the 1960s, the theory of functional decentralisation was used extensively in their defence (Van den Berg 1982; Havekes 2008; Mostert 2017). Moreover, from 1972 onwards the water boards were again presented as the oldest democratic government bodies in the Netherlands (Van Tielhof 2015). This might be a reaction to criticism at the time of functional decentralisation as undemocratic (cf. Van den Berg 1982, pp. 64–73). Since 2014, and after much modernisation, the position of the water boards seems to be secure (Mostert 2017).

## Sense of place

There is one final theme concerning water and the Dutch identity to be discussed, sense of place (e.g. Stedman 2003; Lewicka 2011). Sense of place refers to the symbolic meaning of and emotional attachment to a place. Applied to water, it refers to water as a part of the landscape.

Water, in particular rives and the sea, was an important subject in seventeenth Century Dutch landscape painting. Yet, while realistic in style, the paintings usually had an artificial composition, and typically Dutch subjects such as polders and polder mills were rarely painted (Schapelhouman and Schatborn 1987; Os 2008). This changed around 1860, when painters of the Hague School started to paint polders, lakes, canals and beaches, often with overcast skies. Their paintings were seen as truly national, simple and truthful, and standing in the tradition of seventeenth Century landscape painting (Van Santen Kolff 1875; Korevaar-Hesseling 1941).

Slightly later, around 1880, important changes took place in Dutch poetry as well. There had been depictions of the landscape in Dutch poetry before, but these tended to be quite generic and heavily influenced by ancient mythology or primarily projections of emotions. Around 1880, attention to the landscape itself increased and observation and emotions became one (Van Leeuwen 1941; Donker 1945; Van Toorn 1998). The Netherlands were depicted as a country of “wide rivers flowing slowly through endless lowlands” under a low-hanging sky, where “the voice of the water, with its eternal disasters, is feared and heard” (Hendrik Marsman, “Memory of Holland”, 1936). In addition, the Sea played a major role (Donker 1945, pp. 264–276). While the sky stood for infinity, the rivers for the slow flow of time, and the lowlands for the here and now where the people toil, the Sea stood for eternity. The Sea, “from which (Holland) was born as from a shell” and that “each can hear in his soul” (C.S. Adama van Scheltema, “Holland!”, 1906), and from which it will re-emerge when it floods the country (e.g. M. Revis, “Luctor et Emergo”, 1940), is also the Sea to which it eventually will return. It “pounds the shore in an endless swell” (W. Kloos, “De Zee, de Zee klotst voort in eindeloze deining”, 1894).

Similar sentiments are expressed in some recent popular songs. In “The foundling of Ameland”, written by Freek de Jonge but best known in the version by Boudewijn de Groot from 2004, a baby boy is washed ashore on the Isle of Ameland, tied to a lifebuoy. On the waves, he had felt as in the womb. As an infant, he would stand on the beach until the tide would turn, unafraid of the wild sea, yelling, “I am coming, sea, sun, wind, ocean, I am coming.” One day in the evening sun, he undresses on the beach, starts to yell once more, walks on the water for a moment and then sinks into the depths and drowns.^5^

It is easy to see religious references in these poems and songs, but emotional attachment to water can also be much more down to earth. In 2018, the Social and Cultural Planning Agency conducted a large survey on how Dutch people experienced the Dutch identity (Beugelsdijk et al. 2019). Five thousand persons were asked to react to a list with 185 items and indicate how characteristic each item is for the Netherlands and how much they feel connected with that items. The Dutch language scored highest, but also water-related items scored very high, such as the polder landscape, the Wadden Sea and the Dutch islands, the Harbour of Rotterdam, polder mills, dykes and the Delta works. Between 80 and 90% of the respondents considered these items to be very characteristic for the Netherlands. The scores for connection were lower, but still ranged from 44 to 55%.

**Fig. 2:**
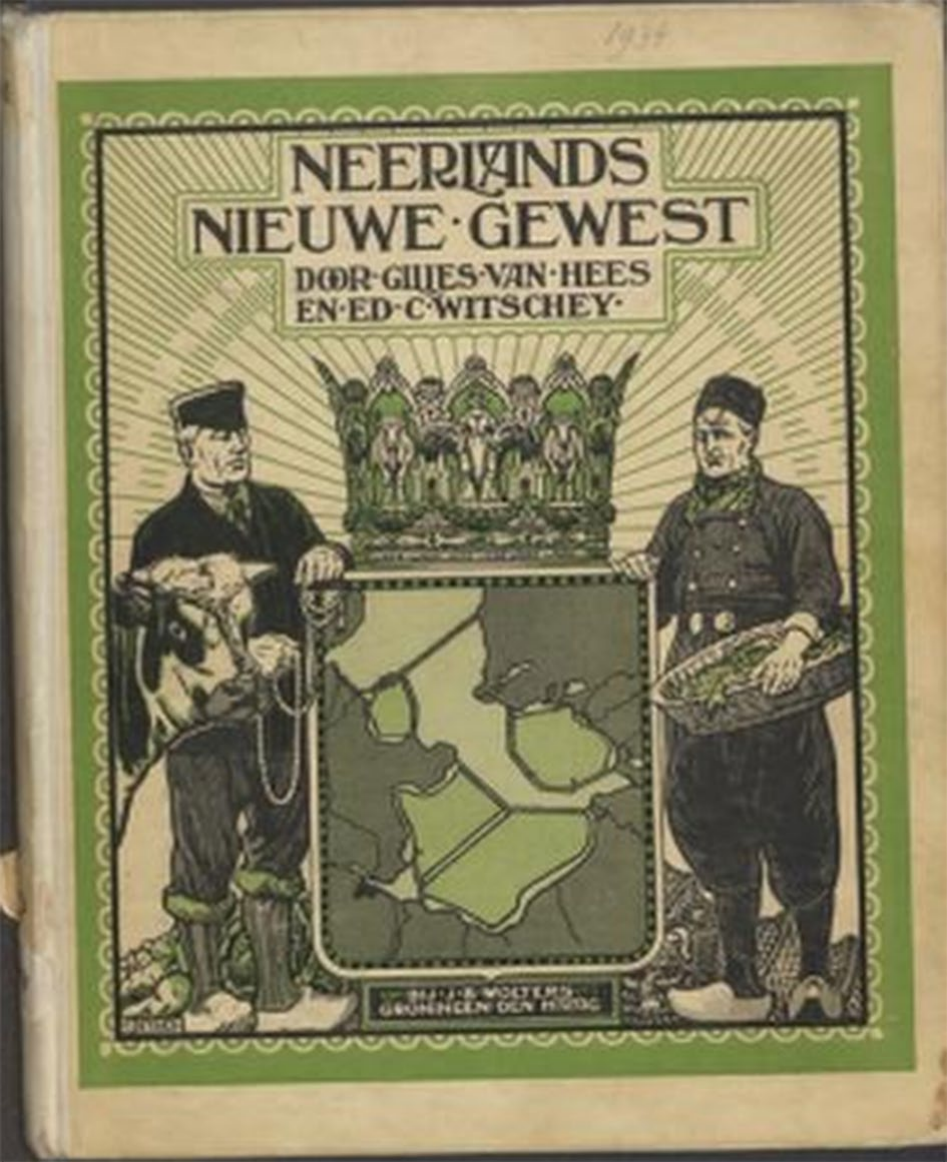
Cover of “Neerlands nieuw gewest” (the Netherland’s new province: Van Hees and Witscheij 1931). The lion in the Dutch coat of arms has been replaced by a map of the Zuiderzee works, which were then under execution. The old-fashioned spelling “Neerlands” harks back to the glorious Dutch past, while the sunrays herald a bright future

The importance of water for the Dutch identity is also reflected in a prize-winning and bestselling picture book for children, titled Nederland (Dematons 2012; Dematons and Goossens 2013). This book contains 26 double-paged pictures of the Netherlands, starting with a picture of the North Sea showing both seventeenth Century and modern ships and closing with a picture of the Afsluitdijk, a 32 km long dam, completed in 1932, that closed off the Zuiderzee, improved flood protection and facilitated large-scale land reclamation (see also Fig. 2). In between, there are depictions of the beach and the dunes, the Wadden Sea area, the Harbour of Rotterdam, the canals of Amsterdam, a steam pumping station, the new-year dip in the sea at Katwijk, the 1953 flood disaster, ice-skating, the main rivers, floating houses, and much more. Most pictures show much water and in all there is at least some. While written for children, the book has also been recommended to read with people suffering from dementia (https://dementie.nl/nieuws/leuke-dingen/gezellige-tips-voor-koningsdag, consulted on 22 April 2020). This suggests it reflects deeply-held images of the Netherlands—deeply-held in the early twenty first Century.

## Discussion

This article discussed the theory that the Dutch identity has been shaped by water. The origin and development of this theory are closely linked with the development of the Netherlands as a nation. The oldest element, the “fight against the water”, predates the Dutch nation by several centuries and initially had a more local or regional character. In 1740–1741, the first national flood relief action was organised, suggesting that by then the Dutch nation was a reality, at least in the eyes of the organisers. Around 1780, land reclamation started to figure prominently in discourses on the nation as a source of national pride and an inspiration for national regeneration. In particular the Patriots and later the liberals emphasised the fact that the Dutch people had “wrung” their country from the water. For orthodox Calvinists water became a national symbol too, but they associated the nation with religion and the leading role of the House of Orange and focused more on flooding. For Roman Catholics and Socialists, water played a smaller role, but by 1900 it was a national symbol for all major social groups, even if their views on the nation differed.

From the late eighteenth Century onwards, the Dutch fight against the water was linked to a number of national characteristics, such as diligence, ingenuity and entrepreneurship. The fight against the water would furthermore have stimulated cooperation, an egalitarian society and democratic institutions, the first being the water boards. The theory of the water boards as the first democratic institutions dates from 1820 and was reinvented in 1972, at a time when their position was challenged. From at least the seventeenth Century onwards, water also played an important role in Dutch imagery as a landscape element, as witnessed by Dutch landscape painting. From at least the nineteenth Century onwards, these images were seen as national, as opposed to only regional or local.

If we define national identity as the self-images of a nation, a key question is who belong to that nation and consequently whose images count. Geographically, the Dutch water identity is skewed towards the low-lying western part of the Netherland, which is also the economic heartland of the country (Huizinga 1935, p. 4; Van Eijndhoven and Leverink 2009; Kennedy 2019). A highly political question concerns the position of immigrants and their descendants. Do only the descendants of the “Fathers” who wrung the country from the sea belong to the nation, everybody born and raised in the Netherlands (e.g. Feith 1793), or everybody currently living in the Netherlands?

According to Renan (1882, p. 9), “the essences of a nation is that all individuals have much in common, and also have forgotten a great many things”. With respect to water and the Dutch nation, the things forgotten include the drainage of peat areas and peat mining. These activities have resulted in land subsidence, the creation of large lakes and increased flooding (e.g. Besteman 1997; Leenders 2004; Van de Ven 2004). This does not fit well in the image of the Dutch as having created their own country. Peat drainage and peat mining are therefore rarely mentioned in books for a broad audience (exceptions: Kruijt 1938, p. 9; Van Eijndhoven and Leverink 2009; Dematons, 2012).

As stated in the introduction, national identities develop in a historical context. In the Netherlands water formed an important part of that context, but it did not determine the national identity completely. According to Leerssen (2011, p. 20), national identities are cultivated to provide certainty in times of political uncertainty, and already in 1942, the Dutch historian Romein noted that most works on the Dutch national character had been written during crises (Romein 1942, pp. 16–17). Whenever there was fast social change, political turmoil or an external threat, as in the late eighteenth Century, the late nineteenth Century, the 1930s and 1940s and since the 1990s, there is discussion on what the nation is or was and should remain or become again. In these discussions the Dutch water history was and is used selectively to stimulate (or simulate) national unity and pride and promote national action.

A clear example of this selective use of history is offered by Van der Houven van Oordt, between 1886 and 1901 secretary of the Zuiderzee-Vereeniging, an NGO promoting the Zuiderzee works (Van der Houven van Oordt 1898; cf. Fig. 2). He argued that, contrary to many MPs, the Dutch people were in favour of these works because they “had always executed such work with courage and gusto, (…) they considered building dykes and land reclamation as their vocation; (and) it is in their character” (p. 165). In addition, the Zuiderzee works would provide a common goal that could unite the nation and would strengthen it economically and morally (pp. 177–178; see also De Pater 2000).

Currently, a large research project, led by Lotte Jensen (Radboud University, Nijmegen), investigates the role of disasters in shaping local and national identities in the Netherlands in the period 1421–1890. In this project the cultural responses in different media (painting, newspapers, etc.) to floods and other disasters are mapped, tracing changes as well as continuities, such as the nation’s struggle against water (https://dealingwithdisasters.nl/en; see also Jensen 2018, 2019; Duiveman 2019; Van Asperen 2020). The analysis in this article suggests that, in as far as water and the national identity are concerned, land reclamation was as important as flood disasters, but in a somewhat different way and for different social groups. In addition, this article identifies a change in the eighteenth Century: before, the nation was still in a nascent state or non-existent, while in the eighteenth Century signs of national solidarity appeared and land reclamation became a source of national pride. A final difference with the research project is that this article covers the period until the present.

The research conducted for this article has been far from exhaustive, and future research can add many details and may require some corrections. First, it would be interesting to analyse cultural representations of land reclamation in the Netherlands in the seventeenth, eighteenth and nineteenth Century in detail, as a complement to the research project led by Lotte Jensen. The proposed period would allow changing representations to be linked to the development of the Netherlands as a nation and nationalism. Secondly, it would be interesting to systematically analyse how Dutch school books on geography and history discussed water in the Netherlands and whether they emphasised floods or land reclamation. This would provide information not only on the dissemination of ideas to broader groups, but also on differences between liberal, orthodox Calvinist and Roman-Catholic discourses since from the nineteenth Century onwards each group had its own schools and school books. Thirdly, it would be interesting to analyse the role that national identity arguments played in discussions on major hydraulic works, such as the reclamation of Lake Haarlem (17th Century–mid nineteenth Century), the Zuiderzee works (mid nineteenth Century–2003) and the Delta works (1930s–1997), and to compare this role with the role of technical and economic arguments. The focus would not be on how important water was for the national identity, but conversely how important the national identity was for water. Finally, it would be interesting to compare the Netherlands with other countries where national development or regeneration has been used as an argument for water resources development, such as Spain.

## Data Availability

Not applicable.

## Footnotes

1 The only reason why no despotic regime developed in the Netherlands would have been the influence of nearby non-hydraulic civilisations (Wittfogel 1957, p. 12).

2 It is impossible to precisely date the Dutch nation as an imagined community at the level of the Netherlands since it developed gradually and not at the same speed in the different regions and social classes. The use of the word “Fatherland” does not prove the existence of a Dutch nation as it often referred to one’s town or region (Meijers Drees 2016).

3 The main political division in the first half of the nineteenth Century was liberalism versus conservatism, but by the end of the century conservatism had disappeared as a separate political current and had become part of confessional politics while there was also a (moderately) conservative current among liberals (the “Old” or “Free Liberals’). Roman Catholics sided politically with the Liberals, but this changed around 1870 because of disagreement over confessional education and anti-liberal papal encyclicals (Oud 1982).

4 The theory was used by Brouwer (1839, p. 3) in a controversy concerning the river polders in the province of Gelderland (see Thorbecke 1843; Korf 1977). Brouwer’s version of the theory remained known in a small circle of water board lawyers until at least the 1960s (e.g. Schepel 1906; Schilthuis 1960).

5 See also the refrain of the similarly popular song “Watermensen” (Water people) by the 3Js from 2007: “Let the water threaten me/ Let it grab me by the throat/ Only for us the water people/ The water will never be too much./ And if the water finally gets us/ Then it has lasted long enough/ Then we the water people/ Will be sent to a higher plan.”.

## References

[CR1] Al–Z J (1783) Brief van een Russischen edelman Iwan Al–z, bevattende eene beschryving van zyn bezoek, afgelegd by den heer John Bertram, beroemd kruidkundige in Pensylvanie, waar in een keurig verslag gegeeven wordt van de huishouding, denk- en leevenswyze diens Americaanschen wysgeers. Vaderl Lett 5:262–268

[CR2] Alutarius H (1656–1657) Gorinchems ys-lijke water-nood, en heug-lijke verlossing: en daar in de bysondere verderf-sonden onses vaderlands, en heilzame remedien daar tegen. P. Vink, Gorinchem

[CR3] Anderson B (2006). Imagined communities: reflections on the origin and spread of nationalism.

[CR4] Andriessen PJ (1874). Feestgeschenk voor onze jongens en meisjes bij het zilvren feest van Z.M. den Koning, 12 mei 1849–1874.

[CR5] Bank J, Van Buuren M (2000). 1900. Hoogtij van burgerlijke cultuur.

[CR6] Been JH (1908). Paddeltje, de scheepsjongen van Michiel de Ruijter.

[CR7] Besteman JC, Boer DEH, Cordfunke EHP, Sarfatij H (1997). Van Assendelft naar Amsterdam. Occupatie en ontginning van de Noordhollandse veengebieden in de middeleeuwen. Holland en het water in de middeleeuwen: strijd tegen het water en beheersing en gebruik van het water.

[CR8] Beugelsdijk S, De Hart J, Van Houwelinge P, Versantvoort M (2019). Denkend aan Nederland; Sociaal en Cultureel Rapport 2019.

[CR9] Bilderdijk W (1832) Geschiedenis des vaderlands. Deel 1. P. Meyer Warnars, Amsterdam

[CR10] Blink H (1896). Nieuwe uitgaven; Een hydrografisch standaardwerk van Noord-Holland. Tijdschr van het Aardrijkskd Genoot Tweede Ser.

[CR11] Borger GJ, Boer DEH, Cordfunke EHP, Sarfatij H (1997). De strijd tegen en om het water: een samenvattende terugblik. Holland en het water in de middeleeuwen: strijd tegen het water en beheersing en gebruik van het water.

[CR12] Bosch T (2012). Natuur en cultuur; Modernisering van hulpverlening na catastrofale overstromingen in de Nederlandse Delta, 1740–1861. Tijdschr voor Waterstaatsgeschied.

[CR13] Brouwer AG (1839). Proeve ten betoog der dringende behoefte van dijk- en polderzaken in Gelderland, aan eene grondwettige herstelling.

[CR14] Brusse MJ (1935). Hou je roer recht: een bundel novellen, impressies, interviews, schetsen, verzen en muziek.

[CR15] Buma WJ, Ebel W (1963). Das Rüstringer Recht Altfriesische Rechtsquellen 1.

[CR16] Committee for the Development of the Dutch Canon (2007). A Key to Dutch History; report by the committee for the development of the Dutch Canon.

[CR17] De Neve RG, Van Heezik AAS, Beukers E (2007). Verbonden door het water; Binnenvaart en zeehavens in Holland. Hollanders en het water: twintig eeuwen strijd en profijt.

[CR18] De Pater B (2000). Vergane glorie, nieuw elan: inpoldering en beelden van de Zuiderzee. Hist geogr tijdschr.

[CR19] De Vries S (1687) Wonderen soo aen als in, en wonder-gevallen soo op als ontrent de zeeën, rivieren, meiren, poelen en fonteynen, historischer, ondersoeckender, en redenvoorstellender wijs verhandeld. Jan ten Hoorn, Amsterdam

[CR20] Dematons C (2012). Nederland.

[CR21] Dematons C, Goossens JM (2013). Duizend dingen over Nederland.

[CR22] Dibbits HAMC (1950). Nederland-waterland: een historisch-technisch overzicht.

[CR23] Donker A (1945). Karaktertrekken der vaderlandsche letterkunde.

[CR24] Donker Curtius D (1834). De regtsmagt der hooge- en andere heemraadschappen betwist.

[CR25] Donker Curtius D (1835). Beantwoording der verdediging van de regtsmagt der heemraadschappen.

[CR26] Duiveman A (2019). Praying for (the) community: disasters, ritual and solidarity in the eighteenth-Century Dutch Republic. Cult Soc Hist.

[CR27] Engelberts (1763) Verdediging van de eer der Hollandsche natie. Yntema en Tiboel, Amsterdam

[CR28] Feith R (1793) Iets over den smaak der Nederlanderen in de Poëzij. In: Feith R (ed) Brieven over verscheidenen onderwerpen; zesde en laatste deel. Johannes Allart, Amsterdam, pp 1–33

[CR29] Fleischer A, Roberts LL, Schaffer S, Dear P (2007). The Beemster Polder: conservative invention and Holland’s great pleasure garden. The mindful hand: inquiry and invention from the late renaissance to early industrialisation.

[CR30] Fruin R, Vissering S (1900) Het karakter van het Nederlandsche volk. In: Blok PJ, Muller PL, Muller Fz S (eds) Robert Fruin’s verspreide geschriften. Deel I Historische opstellen: met aanteekeningen, toevoegsels en verbeteringen uit des schrijvers nalatenschap. M. Nijhoff, ‘s-Gravenhage, pp 1–21

[CR31] Grimme A (1939). De Waterwolf verliest!.

[CR32] Groen van Prinsterer G (1830). Nederlandsche gedachten.

[CR33] Groen van Prinsterer G (1841). Kort overzigt van de geschiedenis des vaderlands.

[CR34] Groen van Prinsterer G (1846). Handboek der geschiedenis van het vaderland.

[CR35] Havekes H (2008). Functioneel decentraal waterbestuur: borging, bescherming en beweging; De institutionele omwenteling van het waterschap in de afgelopen vijftig jaar.

[CR36] Helmers JF (2009). De Hollandsche natie.

[CR37] Hora Siccama J (1848). Neerlands grond en beschaving in verband beschouwd. Alg Lett Maandschr.

[CR38] Huf P (1941). Ons volk: een karakteristiek in verzen.

[CR39] Huizinga J (1935) Nederland’s geestesmerk, Revised edition. Sijthoff, Leiden

[CR40] Huizinga J (1941). Nederland’s beschaving in de zeventiende eeuw: een schets.

[CR41] Janiçon FM (1729) Etat présent de la république des Provinces-Unies et des païs qui en dépendent; Tome premier. Jean van Duren, La Haye

[CR42] Janiçon FM (1731) De Republiek der Vereenigde Nederlanden; Eerste deel. Johannes van Duren, ‘s-Gravenhage

[CR43] Jensen L (2016). De verheerlijking van het verleden: helden, literatuur en natievorming in de negentiende eeuw.

[CR44] Jensen LE (2018). Wij tegen het water; Een eeuwenoude strijd.

[CR45] Jensen L (2019). ‘Disaster upon Disaster Inflicted on the Dutch’. Singing about disasters in the Netherlands, 1600–1900. BMG Low Ctries Hist Rev.

[CR46] Josephus Jitta AC (1932). Functioneele decentralisatie. de grondgedachte van den corporatieven staat binnen het kader der parlementaire democratie.

[CR47] Josephus Jitta AC (1936). De corporatieve staatsgedachte in Nederland.

[CR48] Josephus Jitta AC (1936). Het Nederlandsche volkskarakter.

[CR49] Kennedy J, Kleijn Gd (2019). The art of a nation. Koele wateren.

[CR50] Kesic J, Duyvendak JW (2016). Anti-nationalist nationalism: the paradox of Dutch national identity. Nations Natl.

[CR51] Korevaar-Hesseling EH (1941). Het Nederlandse volkskarakter weerspiegeld in de Nederlandse schilderkunst.

[CR52] Korf J (1977) Het tijdvak van 1838 tot 1954. In: Moorman van Kappen O, Korf J, Verschuer OWAv (eds) Tieler- en Bommelerwaarden 1327–1977; grepen uit de geschiedenis van 650 jaar waterstaatszorg in Tielerwaard en Bommelerwaard. Tiel, pp 235–424

[CR53] Kruijt JP (1934). Het Nederlandse volkskarakter en het socialisme.

[CR54] Kruijt JP (1938). Sociale aardrijkskunde van Nederland voor de hoogste klasse van de Hogere Burgerscholen.

[CR55] Kullberg J, Idema J, Schlette A (2019) Chapter 13, Het Nederlandse landschap en Nederlandse identiteit. In: Beugelsdijk S, De Hart J, Van Houwelinge P, Versantvoort M (eds) Denkend aan Nederland; Sociaal en Cultureel Rapport 2019. Sociaal en Cultureel Planbureau, Den Haag

[CR56] Kuyper A (1879). “Ons program”; (met bijlagen).

[CR57] Last J (1939). Zuiderzee.

[CR58] Lawrence C (2016) The cult of the seventeenth-century Dutch naval heroes: critical appropriations of a popular patriotic tradition. In: Fenoulhet J, Gilbert L (eds) Narratives of Low Countries history and culture: reframing the past. UCL Press, pp 35–43. 10.2307/j.ctt1hd18bd.8

[CR59] Leenders KAHW (2004). De interactie tussen mens en natuur in de strijd om land en water in het zuiden van Holland, 1200–1650. Holl Reg hist tijdschr.

[CR60] Leerssen JT (2011). De bronnen van het vaderland : taal, literatuur en de afbakening van Nederland, 1806–1890.

[CR61] Leerssen J (2016) Imagology: on using ethnicity to make sense of the world. Rev d’études ibér ibéro-am 13–31

[CR62] Lewicka M (2011). Place attachment: how far have we come in the last 40 years?. J Environ Psychol.

[CR63] Meyer JD (1820). Esprit, origine et progrès des institutions judiciaires des principaux pays de l’Europe Tom. IV.

[CR64] Meyer JD (1830) Lezing zonder titel. In: Verslag van de Openbare vergadering der tweede klasse van het Koninklijk-Nederlandsche Instituut van Wetenschappen, Letterkunde en Schoone Kunsten, gehouden in de groote zaal van het Hotel des instituuts op den eersten september des jaars 1830, ‘s voormiddags te elf ure. Pieper en Ipenbuur, Amsterdam, pp 59–78

[CR65] Meijer Drees M (2016) Patriotism in Dutch literature (c. 1650–1750). In: Fenoulhet J, Gilbert L (eds) Narratives of Low Countries history and culture: reframing the past. UCL Press, pp 44–51. 10.2307/j.ctt1hd18bd.8

[CR66] Moens P (1827). Letter-looveren, gestrooid voor mijne jonge landgenooten.

[CR67] Mostert E (2015). Children’s books as a historical source; flooding in 20th century Dutch children’s books. Water Hist.

[CR68] Mostert E (2017). Between arguments, interests and expertise: the institutional development of the Dutch water boards, 1953–present. Water Hist.

[CR69] Multatuli (1877) Ideën, 2nd edn. V. G.L. Funke, Amsterdam

[CR70] Niemeijer AFJ (2016). Wie ‘schiep” Nederland nu echt?. Een reactie op een vrijzinnige gedachte Vitruvius.

[CR71] NN (1818). Galerie historique des contemporains, ou Nouvelle biographie.

[CR72] NN (1874). De Oranjeboom: een feestgeschenk voor de Nederlandsche jeugd, 12 Mei 1874.

[CR73] NN (1950). De schoonheid van ons land.

[CR74] Norel K (1939). Pioniers in Zuiderzeeland.

[CR75] Ockerse WA (1797) Ontwerp tot eene algemeene characterkunde; derde stukjen, behelzende het nationaal character der Nederlanderen. Van Paddeburg en Zoon, Utrecht

[CR76] Oud PJ (1982). Honderd jaren; een eeuw van staatkudige vormgeving in Nederland 1840–1940.

[CR77] Paradijs C (1887). Grassprietjes, of Liederen op het gebied van deugd, godsvruchten en vaderland.

[CR78] Pitcairn A (1727) Selecta Poemata A. Pitcarnii Med. Doctoris, Gulielmi Scot a Thirlestane, Equitis, Thomae Kincadii, Civis Edinburgensis et aliorum. Robert Freebairn, Edinburg

[CR79] Pleij H (2016). Moet nog steeds kunnen – Op zoek naar een Nederlandse identiteit.

[CR80] Potgieter EJ (1833). Holl Vaderl lett.

[CR81] Potgieter EJ (1844) De zusters. De Gids 8:298–336, 350–375, 509–533, 610–650

[CR82] Raedts PGJM (2011). De ontdekking van de Middeleeuwen: geschiedenis van een illusie.

[CR83] Renan E (1882). Qu’est-ce qu’une nation?.

[CR84] Romein J (1934). De lage landen bij de zee: geïllustreerde geschiedenis van het Nederlandsche volk van Duinkerken tot Delfzijl.

[CR85] Romein J (1942). Beschouwingen over het Nederlandse volkskarakte.

[CR86] Rüter AJC (1945). De Nederlandsche natie en het Nederlandsche volkskarater.

[CR87] Schama S (1988) Overvloed en onbehagen: de Nederlandse cultuur in de Gouden Eeuw [trans of idem (1987) The embarrassment of riches: an interpretation of Dutch culture in the Golden Age. Knopf, New York]. Contact, Amsterdam

[CR88] Schapelhouman M, Schatborn P (1987). Land & water: Hollandse tekeningen uit de 17de eeuw in het Rijksprentenkabinet = Dutch drawings from the 17th century in the Rijksmuseum Print Room.

[CR89] Schepel CJH (1906). Waterschapswetgeving.

[CR90] Schilthuis GJC (1960) Waterschapsrecht. 2e dr. edn. Samsom, Alphen aan den Rijn

[CR91] Staverman WH (1946) Het Nederlandsche volkskarakter. Uitgeverij W. van Hoeve, ‘s Gravenhage

[CR92] Stedman RA (2003). Is it really just a social construction? The contribution of the physical environment to sense of place. Soc Nat Resour.

[CR93] Stibbe M (1940). Het Nederlandsche volkskarakter.

[CR94] Sturkenboom D (2014). Understanding emotional identities: the Dutch phlegmatic temperament as historical case-study. BMGN Low Ctries Hist Rev.

[CR95] Stuurman S (1992). Wacht op onze daden: het liberalisme en de vernieuwing van de Nederlandse staat.

[CR96] Sundberg AD (2015) Floods, worms, and cattle plague: nature-induced disaster at the closing of the Dutch Golden Age, 1672–1764. Dissertation, University of Kansas

[CR97] Swyngedouw E (1999). Modernity and hybridity: nature, regeneracionismo, and the production of the Spanish waterscape, 1890–1930. Ann Assoc Am Geogr.

[CR98] Thorbecke JR (1843) Brief aan een lid der Staten van Gelderland, over de magt der Provinciale Staten uit Art. 220 der Grondwet. P.H. van den Heuvell, Leiden

[CR99] Ultrajectinus (1783) Briev van Ultrajectinus, over de onbestaanbaarheid der Recommendatien met de Vroedschaps-Eed te Utrecht. De polit kruyer 2:799–806

[CR100] Van Asperen H (2020). Disaster and discord: Romeyn de Hooghe and the Dutch state of ruination in 1675. Dutch Crossing J Low Ctries Stud.

[CR101] Van de Ven GP (2004). Man-made lowlands; history of water management and land reclamation in the Netherlands.

[CR102] Van den Berg JT (1982). Waterschap en functionele decentralisatie.

[CR103] Van den Brink G (2018). Waartoe is Nederland op aarde?.

[CR104] Van den Vondel J (1930) De werken van Vondel. Deel 4. 1640–1645. Maatschappij voor goede en goedkoope lectuur, Amsterdam

[CR105] Van den Vondel J (1987). Twee zeevaart-gedichten.

[CR106] Van den Vondel J (1994). Gysbreght van Aemstel.

[CR107] Van der Ham W, Jacobs I (2004). Hoge dijken, diepe gronden. Land en water tussen Rotterdam en Gouda: een geschiedenis van Schieland.

[CR108] Van der Houven van Oordt HC (1898). De economische beteekenis van de afsluiting en drooglegging der Zuiderzee.

[CR109] Van der Pot CW, Donner AM (1977). Handboek van het Nederlandse staatsrecht.

[CR110] Van Eijndhoven JCM, Leverink R (2009). Zoden aan de dijk : 25 peilingen naar Nederland als waterland.

[CR111] Van Haren OZ (1776) De Geusen; proeve van een vaderlands gedicht, 3rd edn. Simon Clement, Zwolle

[CR112] Van Heerikhuizen B (1980). Sociologen in de jaren dertig en veertig over het Nederlandse volkskarakter. Amst Sociol Tijdschr.

[CR113] Van Hees G, Witscheij EC (1931). Neerlands nieuwe gewest: leer-leesboek voor de hoogste klasse der lagere school, vervolg- en meer uitgebreid lager onderwijs.

[CR114] Van Leeuwen WLME (1941). De liefde tot zijn land is ieder aangeboren: land en volk van Nederland in kunst en letteren.

[CR115] Van Lennep DJ (1828) Verhandeling over het belangrijke van Hollands grond en oudheden voor gevoel en verbeelding. s.p., s.l.

[CR116] Van Os HW (2008). De ontdekking van Nederland: vier eeuwen landschap verbeeld door Hollandse meesters.

[CR117] Van Santen Kolff J (1875) Een blik in de Hollandsche schilderschool onzer dagen; IV. De banier; tijdschr van “Het jonge Holland” 1:157–203

[CR118] Van Sas NCF (2005). De metamorfose van Nederland; Van oude orde naar moderniteit, 1750–1900.

[CR119] Van Schendel AFE (1945). De Nederlanden: een gedicht.

[CR120] Van Tielhof M (2015). Waterschappen als de oudste democratische instellingen van Nederland; Het ontstaan van een mythe. Tijdschr voor Waterstaatsgeschied.

[CR121] Van Toorn WP (1998). Leesbaar landschap.

[CR122] Vandersmissen H, Bos H, Burlage A, Hoogers M, Nijland R (1995). Watersnood 1995; Nationaal aktieboek.

[CR123] Voorregt AM (1784) Briev van A.M. Voorregt over het gedrag van zekere Heemraden in ‘t Land van Altena, en het regt der Ingelanden tot verkiezing van dezelve. De polit kruyer 3:1779–1781

[CR124] Vuijsje H, Van der Lans J (1999). Typisch Nederlands; Vademecum van de Nederlandse identiteit.

[CR125] Werumeus Buning JWF (ed) (1940) Lof van Nederland : zijnde een verzameling zoowel van ouds beproefde als kortelings geschreven gedichten, verzen en rijmen waarin de schoonheid des vaderlands te water en te land wordt zichtbaar gemaakt mitsgaders eenige beschrijvingen van de maan en van schoone bloemstukken, vrouwspersonen en andere aanverwante zaken. Querido, Amsterdam

[CR126] Wittfogel KA (1957) Oriental despotism; a comparative study of total power. Yale University Press, New Haven, Oxford University Press, London

[CR127] Wolff B, Deken A (1784) Historie van den heer Willem Leevend. Deel 1. Isaac van Cleef, Den Haag

